# Arthroplasty versus internal fixation for femoral neck fractures in the elderly

**DOI:** 10.1007/s11751-010-0099-3

**Published:** 2011-01-14

**Authors:** Vassilios Nicolaides, Spyridon Galanakos, Andreas F. Mavrogenis, Vasileios I. Sakellariou, Ioannis Papakostas, Constantinos E. Nikolopoulos, Panayiotis J. Papagelopoulos

**Affiliations:** 1 Fourth Department of Orthopaedics, KAT Hospital, 63 Magnisias str, Dionysos, Attiki, 14576 Athens, Greece; 2 First Department of Orthopaedics, Athens University Medical School, 41 Ventouri Street, 15562 Holargos, Athens, Greece; 3 Third Department of Orthopaedics, KAT Hospital, Athens, Greece

**Keywords:** Femoral neck fracture, Arthroplasty, Internal fixation, Harris hip score

## Abstract

We studied 140 patients with femoral neck fractures treated from January 1999 to December 2006. There were 68 men and 72 women with a mean age of 72 years (range 60–80 years). Seventy patients were treated with closed reduction and internal fixation (group A), and 70 patients with hip arthroplasty (group B). The duration of surgery, length of hospitalization, complications, postoperative Harris hip score, and need for reoperation were recorded. Group B had significantly higher blood loss, increased surgical time and length of hospitalization compared to group A patients. The Harris hip score was significantly higher in group B at the 3, 6, and 12-month follow-up evaluations; however, the differences were no longer significant at the 24-month evaluation. The overall complications rate was 18.6% (13 patients) in group A compared to 25.7% (18 patients) in group B; this was not statistically significant (*P* = 0.309). A statistically significant difference was found regarding reoperation rate in group A (11.4%, eight patients) compared to group B (1.4%, one patient) (*P* = 0.016). Arthroplasty compared to internal fixation for displaced femoral neck fractures is associated with a significantly higher functional score and lower risk of reoperation at the cost of greater infection rates, blood loss, and operative time.

## Introduction

The incidence of proximal femoral fractures has increased significantly in recent years and is expected to continue to rise with increasing life expectancy of elderly patients [1]. The management of intracapsular hip fractures in the elderly, in general, includes resection of the femoral head and hip replacement [2]. In contrary, for patients younger than 60 years, preservation of the femoral head and anatomic reduction and stable fixation of a femoral neck fracture is the main concern [3]. However, in patients aged between 60 and 80 years, the decision between internal fixation and arthroplasty remains controversial [2]. In this age group, the optimal treatment should be individualized depending on the fracture pattern and displacement, and preoperative ambulation, level of independence, disability and general health status of the patients [4–6].

The aim of the present study was to evaluate the functional outcome and quality of life of patients aged 60–80 years with femoral neck fractures treated with hip arthroplasty or closed reduction and internal fixation.

## Patients and methods

We retrospectively analyzed the medical records of 140 elderly patients admitted at the authors’ institution for a femoral neck fracture in the time period from January 1999 through December 2006. There were 68 men and 72 women with a mean age of 72 years (range 60–80 years). The patients were allocated into two groups: group A included 70 patients treated with closed reduction and internal fixation with cannulated hip screws (Smith and Nephew/Memphis, Tennessee), and group B included 70 patients treated with a hip arthroplasty (Synergy, Smith and Nephew/Memphis, Tennessee). Patients of both groups were matched according to age and gender, and preoperative medical and physical condition, ambulation ability, and co-morbidities (Table 1). All patients gave written informed consent to be included in this study. The study has been approved by the institutional review board/ethics committee of the authors’ institutions.

**Table 1:** Details of the patients included in this study

	Group A (internal fixation)	Group B (arthroplasty)
Mean age (years)	64	72
Gender (M/F)	32/38	36/34
Mechanism of injury		
Simple fall	42	66
Fall from height	23	4
Motor vehicle accident	5	0
Type of fracture	Garden type I–IV	Garden type III and IV
Mean preoperative Hb (g/dl)	12.4	11.3

At the same time period, 1,248 patients with femoral neck fractures were admitted at the authors’ institutions. Inclusion criteria for this study were acute, intracapsular femoral neck fractures (Garden types I–IV), age between 60 and 80 years, and independent ambulation before the injury. From these patients, 140 patients met these criteria and were included in this study. The remaining 1,108 patients had basocervical/extracapsular hip fractures, bilateral hip fractures, pathological hip fractures, previous ipsilateral hip fracture or hip surgery, significant co-morbidities, and intensive care or treatment in medical departments, or had deceased at the latest examination, and were excluded.

The decision between closed reduction and internal fixation was based on the age of the patients and preoperative medical and physical condition, ambulation ability, and co-morbidities; in patients with good general health status and Garden types I–IV femoral neck fractures, closed reduction, if necessary, and internal fixation was performed (group A); if closed reduction was not possible or comminution was observed, total hip arthroplasty was performed (group B). In patients with poor general health status and Garden types III and IV femoral neck fractures, hip hemiarthroplasty was performed (group B).

In group A patients, closed reduction and internal fixation with cannulated screws was done according to Lindequist and Törnkvist [7]. In group B, a cementless femoral prosthesis was inserted in 37 patients (52.8%) and a cemented femoral prosthesis in 33 patients (47.2%) depending on intraoperative findings of poor bone quality; in all total arthroplasty patients, a cementless cup was inserted. In all group B patients, a Hardigne approach has been used [8], and a bipolar prosthetic femoral head was inserted in the hemiarthroplasty procedures. All patients received perioperative antibiotics prophylaxis with second-generation cephalosporin and anticoagulation prophylaxis with low molecular weight heparins.

The postoperative mobilization protocol included immediate mobilization starting from the second postoperative day with partial weight bearing as tolerated with the use of crutches or a walker for 6 weeks and then full weight bearing. The arthroplasty patients were instructed for precautions to avoid dislocation of the prosthesis. The length of hospitalization was determined individually in every patient depending primarily on postoperative general medical status and ability to stand or ambulate with a walker; function was not evaluated at the time of discharge from hospital.

Perioperative data including duration of surgery, need for allogenic blood transfusion, length of hospitalization, and complications were recorded. Postoperative evaluation was done at regular intervals of 3, 6, 12 months and at the latest examination for the purpose of this study. Functional evaluation was done according to the Harris hip score [9] in both groups.

Statistical analysis was done with the paired *t* test using the SPSS software version 15 (SPSS Inc, Chicago Ill.) for each parameter examined (blood transfusion, duration of surgery, length of hospitalization, complications, and Harris hip score).

## Results

The mean follow-up was 6.5 years (range 2.5–10 years). At the latest evaluation, all patients were alive. The mean surgical time for group A was statistically significantly lower (mean 70 min; range 50–110 min; 95% CI 67.66–72.34) compared to group B (mean 100 min; range 70–150 min; 95% CI 95.31–104.69) (*P* = 0.028). The mean blood loss for group A was statistically significantly lower (mean 1.6 blood units; range 1–2 blood units; 95% CI 1.48–1.72) compared to group B (mean 2.8 blood units; range 2–3 blood units; 95% CI 2.66–2.94) (*P* = 0.034). The length of hospitalization was statistically significantly shorter in group A (mean 4.8 days; range 4–6 days; 95% CI 4.57–5.03) compared to group B (mean 5.9 days; range 5–8 days; 95% CI 5.69–6.11) (*P* = 0.049).

**Fig. 1:**
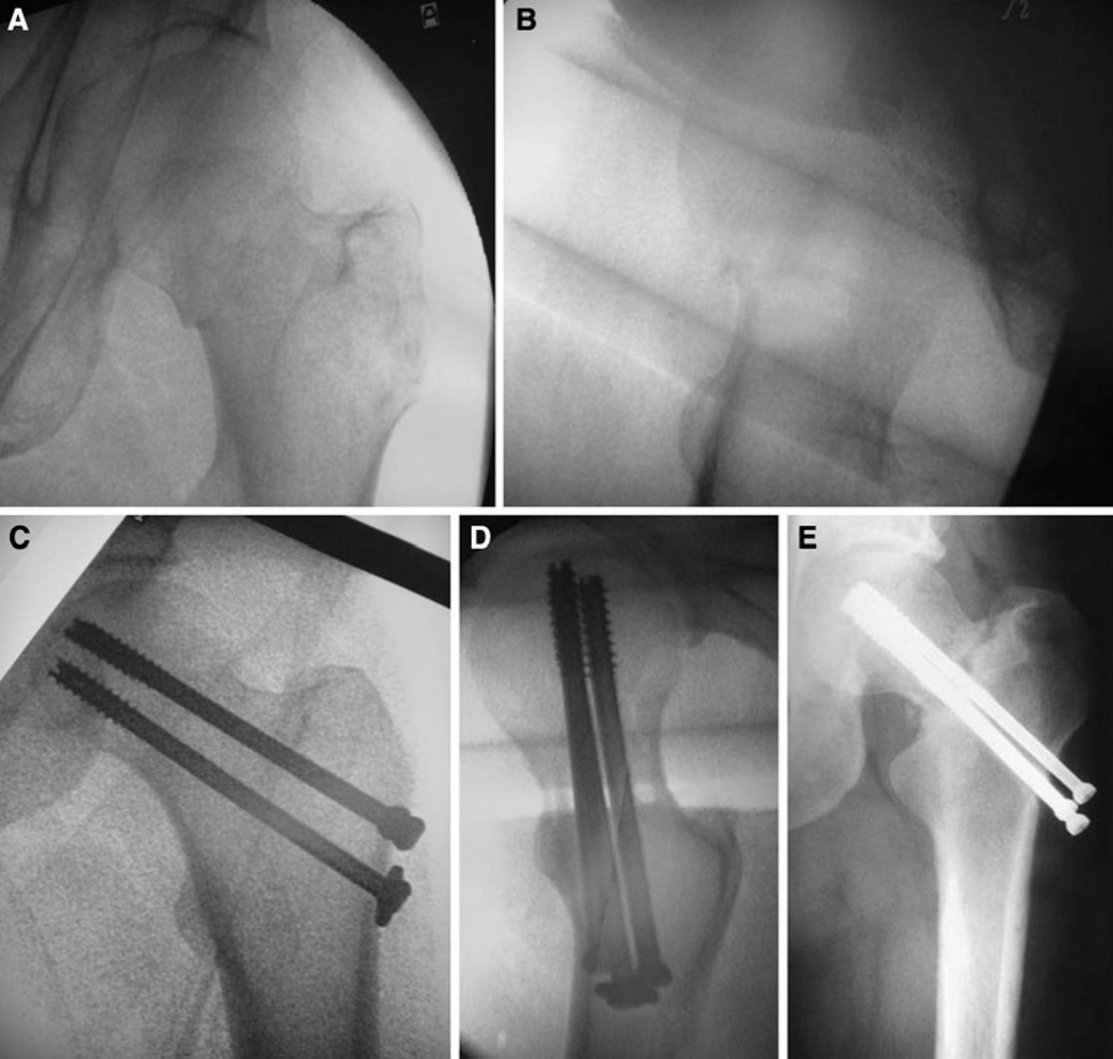
Intraoperative **a** anteroposterior and **b** lateral fluoroscopy views show a non-displaced Garden II femoral neck fracture in a 64-year-old woman. Intraoperative **c** anteroposterior and **d** lateral fluoroscopy views after internal fixation with cannulated hip screws. **e** At 8 months, anteroposterior radiograph of the hip of the patient shown in Fig. 1 shows resorption of the femoral neck and lateral migration of the hip screws, suggesting non-union of the femoral neck fracture. Revision to total hip arthroplasty was done

**Fig. 2:**
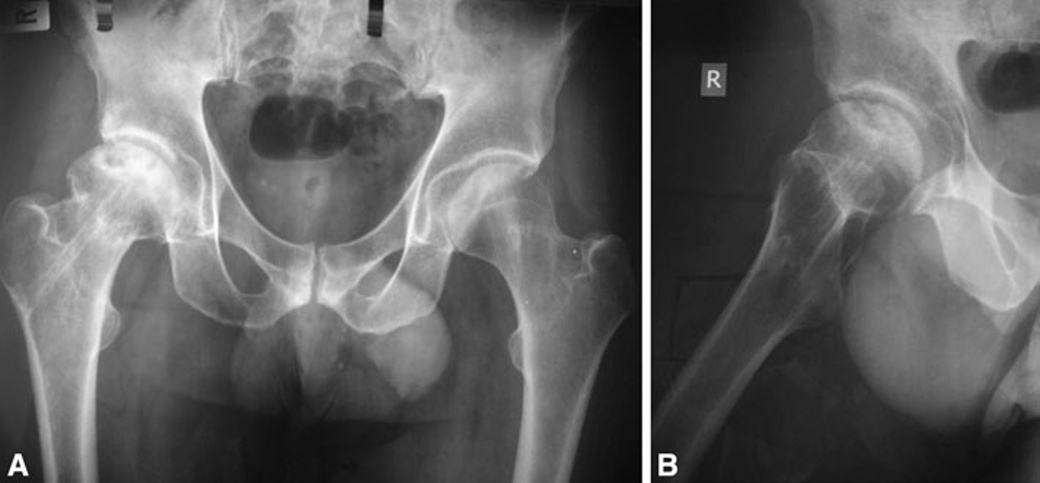
**a** Anteroposterior and **b** lateral radiographs of the right hip joint show avascular necrosis of the femoral head in a 70-year-old man 3.5 years after closed reduction and internal fixation for a Garden type III femoral neck fracture

The overall complications rate was 18.6% (13 patients) in group A compared to 25.7% (18 patients) in group B; this was not statistically significant (*P* = 0.309). More specifically, in group A, delayed union occurred in five patients, non-union in two patients (Fig. 1a–e) and avascular necrosis of the femoral head in six patients (Fig. 2). In group B, acetabular protrusion of 6–10 mm occurred in three patients and severe protrusion of 14 mm in one patient with hip hemiarthroplasty, dislocation treated with closed reduction in three patients with total hip arthroplasty, superficial wound infection in seven patients, and deep venous thrombosis in four patients.

A statistically significant difference was found regarding the need for reoperation in group B compared to group A (*P* = 0.016). Eight group A patients (11.4%) with non-union and avascular necrosis of the femoral head were revised to total hip arthroplasty compared to one group B patient (1.4%) with severe acetabular protrusion also to total hip arthroplasty.

The Harris hip score at 3, 6, and 12 months postoperatively was statistically significantly higher in group B compared to group A (*P* = 0.028, 0.034, and 0.045, respectively at each follow-up evaluation). However, at 24 months, there was no statistically significant difference (*P* = 0.087) between the two groups (Table 2).

**Table 2:** The Harris hip scores at 3-, 6-, 12-, and 24-month follow-up

	3 months			6 months			12 months			24 months		
Harris hip score	Internal fixation	Hemiarthroplasty	Total arthroplasty	Internal fixation	Hemiarthroplasty	Total arthroplasty	Internal fixation	Hemiarthroplasty	Total arthroplasty	Internal fixation	Hemiarthroplasty	Total arthroplasty
Total score	77.3 (48–96)	79.2 (47–95)	80.3 (48–97)	82.7 (50–96)	83.6 (50–96)	85.8 (51–98)	91.8 (53–100)	84.7 (52–97)	94.9 (55–100)	94.8 (54–100)	87.7 (51–97)	95.9 (55–100)
Pain	41 (20–43)	40 (19–42)	40 (19–43)	42 (30–43)	43 (29–43)	43 (30–44)	43 (31–44)	43 (30–44)	44 (33–44)	44 (33–44)	44 (30–44)	44 (33–44)
Function	28 (12–45)	31 (14–45)	32 (15–46)	32 (15–46)	32 (14–46)	34 (18–47)	40 (2547)	33 (20–47)	42 (27–47)	42 (27–47)	35 (20–47)	43 (27–47)
Absence of deformity	4 (4.0)	4 (4.0)	4 (4.0)	4 (4.0)	4 (4.0)	4 (4.0)	4 (4.0)	4 (4.0)	4 (4.0)	4 (4.0)	4 (4.0)	4 (4.0)
Range of motion	4.3 (3.5–5)	4.2 (3.3–5)	4.3 (3.5–5)	4.7 (3.8–5)	4.6 (3.7–5)	4.8 (3.8–5)	4.8 (3.8–5)	4.7 (3.4–5)	4.9 (3.9–5)	4.8 (3.9–5)	4.7 (3.4–5)	4.9 (3.9–5)
	*P* = 0.028			*P* = 0.034			*P* = 0.045			*P* = 0.087		

## Discussion

Surgical treatment of femoral neck fractures is one of the most common procedures performed by orthopedic surgeons. However, the optimal treatment option of displaced femoral neck fractures remains a matter of debate [10–15]. Current treatment options include reduction and internal fixation, hemiarthroplasty, or total hip arthroplasty [3]. Numerous studies have provided evidence for better outcomes after arthroplasty when compared with internal fixation in terms of overall functional scores, abductor muscles function, independent ambulation without walking aids, and quality of life [16–35]. In the present study, we evaluated the treatment of femoral neck fractures in elderly patients using closed reduction and internal fixation with cannulated hip screws compared to hip arthroplasty. Although perioperative blood loss, length of hospitalization, and complications were statistically significantly lower in the internal fixation group of patients, the postoperative functional scores up to the 12-month evaluation and need for reoperation were in favor of the arthroplasty group of patients.

The retrospective design, the lack of a scientifically robust method of randomization, the treatment decision, although not arbitrary, by the treating surgeons, and the combined group of cemented and cementless hemiarthroplasty techniques may be considered limitations of this study. However, the mid- to long-term follow-up, the number of the patients included at the latest examination, and the scoring system increase the value of this study.

Arthroplasty as a mode of treatment of displaced femoral neck fractures in comparison with internal fixation is associated with a significantly lower risk of revision surgery, at the cost of higher infection, blood loss, and surgical time rates [17, 19, 25–28]. Keating et al. [17] recently compared reduction and internal fixation to hemiarthroplasty and total hip arthroplasty in patients older than 60 years of age with displaced femoral neck fractures. In their study, at the 2-year follow-up, the rate of reoperation in the internal fixation group was 39% compared to 5% and 9% in the hemiarthroplasty and total hip arthroplasty groups, respectively. Additionally, the internal fixation group had worse functional and quality of life outcome scores compared with the arthroplasty groups [17]. Others [36] also reported that among people beyond 60 years of age, arthroplasty is associated with better functional outcome, higher health-related quality of life and more independence compared with internal fixation. In the present study, the functional outcome was significantly higher in the arthroplasty group at the 12-month evaluation; however, at the 24-month evaluation, no statistically significant difference was observed between the two groups.

Although by choosing arthroplasty over internal fixation the surgeon effectively eliminates the risks of non-union, malunion, and avascular necrosis of the femoral head, a new set of complications is introduced including infection, prosthetic hip joint dislocation, acetabular protrusion, femoral stem loosening, and thigh pain [16–18]. In the present study, in group B, seven patients developed superficial wound infection, four patients had deep venous thrombosis, three patients had postoperatively dislocation, and four patients had acetabular protrusion. However, at the latest evaluation, reoperation to total hip arthroplasty was necessary in only one patient with severe acetabular protrusion. Based on the literature, there is no consensus regarding the use of bipolar rather than unipolar prosthetic femoral head for hip hemiarthroplasty. Since the most important factors leading to acetabular cartilage erosion and protrusion are age, activity level, and length of follow-up, unipolar hemiarthroplasty is generally recommended in older patients who are less active and have a shorter life expectancy [18, 19]. However, in the present study, this could not be evaluated as a bipolar head has been used in all hemiarthroplasty patients; in these patients, the rate of acetabular protrusion was 5.7%.

Total hip arthroplasty is currently an accepted treatment option for the active elderly patient with a displaced femoral neck fracture [20]. The longevity of total hip arthroplasty, especially in younger, more active patients, has been questioned. In a study [21], 37 patients with a mean age of 70 years or younger with no evidence of acetabular disease treated with primary total hip arthroplasty for a femoral neck fracture were reviewed. At a mean follow-up of 56 months (range 12–112 months), 18 patients (49%) had undergone or were awaiting for a revision surgery. In their study, the authors recommended against primary total hip arthroplasty for displaced femoral neck fracture in the younger patient population without pre-existing hip disease [21]. Others [22, 23] concluded that total hip arthroplasty is the best treatment option for active patients with a longer life expectancy. Their results are in accordance with the results of the present study regarding the rate of reoperation in patients with displaced femoral neck fractures treated with primary total hip arthroplasty.

Compared to hip hemiarthroplasty, total hip arthroplasty is associated with a higher rate of complications and improved functional results and long-term survival of the prostheses [17]. In addition, we consider that the lower reoperation rate in arthroplasty patients outstands the higher cost in this group of patients with femoral neck fractures because of the more expensive implants, and the higher surgical time and length of hospitalization.

## Conclusion

In line with the literature, we suggest hip arthroplasty for elderly patients with a displaced femoral neck fracture. The present study showed that hip arthroplasty compared to internal fixation for the treatment of displaced femoral neck fractures significantly reduces the risk of reoperation at the cost of higher superficial infection, blood loss, operative time, and length of hospitalization rate. Furthermore, postoperative function as evaluated by the Harris hip score was significantly higher in the arthroplasty compared to the internal fixation group up to the 12-month evaluation; however, this difference diminishes at the 24-month evaluation.
